# Characterization of interaction of magnetic nanoparticles with breast cancer cells

**DOI:** 10.1186/s12951-015-0073-9

**Published:** 2015-02-26

**Authors:** Macarena Calero, Michele Chiappi, Ana Lazaro-Carrillo, María José Rodríguez, Francisco Javier Chichón, Kieran Crosbie-Staunton, Adriele Prina-Mello, Yuri Volkov, Angeles Villanueva, José L Carrascosa

**Affiliations:** Departamento de Biología, Universidad Autónoma de Madrid, Cantoblanco, 28049 Madrid, Spain; Department of Macromolecular Structure, Centro Nacional de Biotecnología, Consejo Superior de Investigaciones Científicas, 28049 Madrid, Spain; Department of Clinical Medicine, Trinity Centre for Health Science, James’s Street, Dublin, 8 Ireland; Centre for Research on Adaptive Nanostructures and Nanodevices (CRANN), and AMBER Centre, Trinity College Dublin, College Green, Dublin, 2 Ireland; Instituto Madrileño de Estudios Avanzados en Nanociencia (IMDEA Nanociencia), Cantoblanco, 28049 Madrid, Spain

**Keywords:** MCF-7 cells, Superparamagnetic iron oxide nanoparticles, Intracellular trafficking, Transmission electron microscopy, Cellular uptake, Endocytosis, Cytotoxicity

## Abstract

**Background:**

Different superparamagnetic iron oxide nanoparticles have been tested for their potential use in cancer treatment, as they enter into cells with high effectiveness, do not induce cytotoxicity, and are retained for relatively long periods of time inside the cells. We have analyzed the interaction, internalization and biocompatibility of dimercaptosuccinic acid-coated superparamagnetic iron oxide nanoparticles with an average diameter of 15 nm and negative surface charge in MCF-7 breast cancer cells.

**Results:**

Cells were incubated with dimercaptosuccinic acid-coated superparamagnetic iron oxide nanoparticles for different time intervals, ranging from 0.5 to 72 h. These nanoparticles showed efficient internalization and relatively slow clearance. Time-dependent uptake studies demonstrated the maximum accumulation of dimercaptosuccinic acid-coated superparamagnetic iron oxide nanoparticles after 24 h of incubation, and afterwards they were slowly removed from cells. Superparamagnetic iron oxide nanoparticles were internalized by energy dependent endocytosis and localized in endosomes. Transmission electron microscopy studies showed macropinocytosis uptake and clathrin-mediated internalization depending on the nanoparticles aggregate size. MCF-7 cells accumulated these nanoparticles without any significant effect on cell morphology, cytoskeleton organization, cell cycle distribution, reactive oxygen species generation and cell viability, showing a similar behavior to untreated control cells.

**Conclusions:**

All these findings indicate that dimercaptosuccinic acid-coated superparamagnetic iron oxide nanoparticles have excellent properties in terms of efficiency and biocompatibility for application to target breast cancer cells.

**Electronic supplementary material:**

The online version of this article (doi:10.1186/s12951-015-0073-9) contains supplementary material, which is available to authorized users.

## Background

Although huge efforts have led to worldwide advances in cancer treatment, this multifactorial and heterogeneous disease is still one of the major causes of death in developed countries [1,2]. In the recent years, several reports have focused on the potential use of superparamagnetic iron oxide nanoparticles (SPION) in cancer research. These reports have raised great expectations because SPION are a promising tool for biomedical applications, including diagnosis by magnetic resonance imaging (MRI) and targeted therapy of cancer by hyperthermia and/or releasing anti-cancer molecules, which can be combined in theranostic approaches [3-5].

Factors such as size, shape and surface charge of nanoparticles (NPs) can determine their cellular internalization and distribution and, thus, their effective performance [6,7]. Furthermore, colloidal stability can be achieved, which is essential to ensure reproducibility, as well as to influence the amount of cellular loading and toxicity. The possibility to modify the surface of these particles with biologically active compounds enables transport of therapeutic agents into specific target cells, increasing specificity and avoiding the access of cytotoxic agents to non-target tissues during the delivery process [8]. Different SPION have been tested for potential use in cancer treatment by hyperthermia, as they enter into cells with high effectiveness and without any cytotoxicity, and they are retained for relatively long periods of time inside the cells [3]. The evaluation of the potential use of these nanoparticles requires a precise knowledge of surface modified SPION internalization mechanisms at the ultrastructural level and resulting intracellular pathways, as well as on the fate of SPION inside the cells. Factors such as uptake rate and internalization dynamics are the key to understand how an insufficient cellular accumulation of nanoparticles can lead to usage limitations, for example as imaging probes [9].

In the past few years, there has been a great interest in applying nanotechnology for biomedical studies, in particular for diagnostic and therapeutic purposes. However, the possible toxicity of nanoparticles to humans and environment has become a question of absolute priority in Nanomedicine [4-6,10].

In this regard, cell cultures are important first line tools to screen therapeutic efficiency and safety of drugs (nanoparticles included) and provide essential information to understanding cell-nanoparticle interactions*,* before moving to *in vivo* analysis [11]. Hence, any new magnetic nanoparticle formulation with potential biomedical applications should be accompanied by a detailed study that ensures both its effectiveness and safety. In this sense, several specific parameters and experimental protocols for assessing nanomaterial toxicity have been developed [10].

We have studied the interaction of dimercaptosuccinic acid-coated superparamagnetic iron oxide nanoparticles (DMSA-SPION) with breast cancer cells (MCF-7) in culture. Monodisperse nanoparticles (around 15 nm in diameter) with a high saturation magnetization value, were surface modified by *meso*-2,3-dimercaptosuccinic acid (DMSA) to ensure their dispersion and stability in aqueous buffers and media [12]. Interaction, uptake of the particles (0.05-0.4 mg ml^−1^), as well as their accumulation and persistence inside cells after prolonged incubation (up to 72 h), were assessed by combining optical light and electron microscopy methods. This approach allowed us to correlate the overall cell visualization with the precise localization of SPION inside the cell, their relationship to cell organelles and the analysis of particle shapes and sizes. Furthermore, several cytotoxicity assays, including cell morphology, analysis of cytoskeleton and adhesion proteins, cell cycle distribution, measurement of intracellular reactive oxygen species (ROS) levels and two viability tests, have been carried out to evaluate biocompatibility of these nanoparticles.

## Results and discussion

### DMSA-SPION uptake and internalization in cultured cells

Size, shape and charge of iron oxide nanoparticles, as well as cell type, are important parameters which affect effective internalization of nanoparticles into cells in culture [13-16]. It has been well documented that positively charged magnetic nanoparticles (MNP) showed a higher degree of internalization than neutral and negatively charged MNP due to their effective attachment to negatively charged cell-membrane surface [3,14,16]. Although there are somewhat contradictory findings about cytotoxicity levels between positively or negatively charged nanoparticles [3,17-19], the latter ones are favored due to their overall lower toxicity levels.

**Figure 1:**
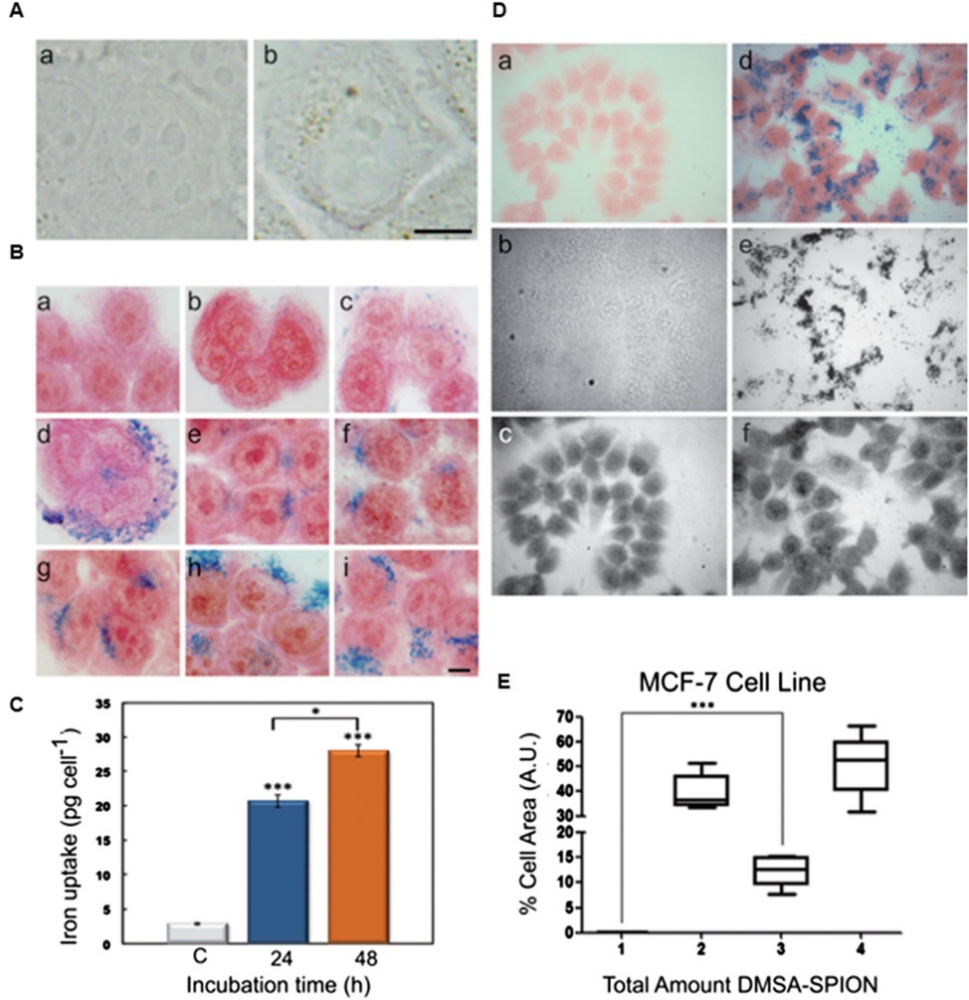
**Uptake and accumulation of DMSA-SPION into cells. (A)** MCF-7 living cells visualized by bright field microscopy. (a) Control cells. (b) Cells incubated with 0.4 mg ml^−1^ SPION for 24 h. Scale bar represents 10 μm. **(B)** Cells incubated with 0.4 mg ml^−1^ SPION for different time, stained with Prussian blue reaction and visualized by bright field microscopy. (a) Control cells. (b-i) Cells incubated for 0.5, 1, 3, 6, 12, 24, 48 and 72 h, respectively. Scale bar represents 10 μm. **(C)** Intracellular iron content quantification by ferrozine assay (expressed as weight of iron per cell), after 24 and 48 h of incubation. **(D, E)** Untreated and incubated MCF-7 (area, red filter), cell with DMSA-SPION. Representative images **(D)** and quantitative box-plot of 100 cells per treatment **(E)**. Details of x-axis: 1) Untreated cell only (background red filter), 2) Untreated, cell only (blue filter), 3) Cell + SPION (total SPION), 4) Cell + SPION (total cell area, blue filter).

Incorporation of DMSA-SPION into MCF-7 cells can be followed by bright field microscopy after 24 h incubation (Figure 1A), where SPION are observed inside living cells, distributed as brown cytoplasmic spots of different sizes, always outside of the nucleus. Similar results have been previously described for iron oxide nanoparticles with different coatings and different sizes in HeLa (human cervical adenocarcinoma) cell line [3,17].

In depth qualitative and quantitative studies on the internalization of DMSA-SPION in MCF-7 cancer cells were performed by both Prussian blue staining and ferrozine-based assay. Figure 1B shows cells incubated with DMSA-SPION for different times (0.5-72 h) by Prussian blue staining. An increase of intracellular DMSA-SPION accumulation was visualized as blue cytoplasmic granular stain within cells directly correlating with incubation times. However, the uptake of nanoparticles seems to reach a saturation point at 24 h. It is important to note that 100% cell labeling efficiency (Prussian blue positive staining) was achieved after 12 h nanoparticles incubation.

These results were confirmed by colorimetric ferrozine-based assay, a widely recognized test to quantify iron in cultured cells [20]. Figure 1C shows intracellular iron concentrations after 24 and 48 h incubation at 0.4 mg ml^−1^ DMSA-SPION (20.67 pg cell^−1^ and 28 pg cell^−1^, respectively). There is abundant literature with regard to SPION-labeling efficiency, although results are difficult to compare because the experimental protocols are different (size and surface coating of the SPION, incubation time, concentration, cell line type, etc.). Generally, prolonged incubation times, as well as elevated iron doses enable to reach higher intracellular loading of SPION and increase labeling efficiency [21,22]. However, overexposure to high concentrations of SPION for extended times may cause cytotoxicity [23]. Therefore, sufficient intracellular uptake of nanoparticles for efficient diagnosis and/or treatment must be balanced with their biocompatibility [17]. In this sense, our results with ferrozine assay indicate that DMSA-SPION accumulate effectively (20.67 pg cell^−1^) within MCF-7 cells. Previously, we had detected 37.1 pg cell^−1^ (into HeLa cells), after 24 h of incubation at 0.5 mg ml^−1^ DMSA magnetic nanoparticles with lower core diameter (9 nm). The small difference in the amount of accumulated iron could be either due to different of SPION diameters (15 *vs* 9 nm) or to the type of cell line (HeLa *vs* MCF-7) [17]. Much lower amounts (5.3 ± 1.1 pg cell^−1^) have been detected over 48 h of incubation with SPION (Feridex®) at 0.075 mg ml^−1^ in labeled NPC (neural progenitor cells) [24].

Quantitative and statistical population analysis of total iron oxide area per total cell area of 100 MCF-7 cells was carried out by automated epifluorescence imaging with multichannel acquisitions (bright field, blue and red channels). From the overlapping and thresholding against the iron content it was possible to identify and quantify the ratio of inorganic iron content versus the total cell area. Figure 1D shows representative microscopy images of untreated and exposed MCF-7 cells to 0.4 mg ml^−1^.

**Figure 2:**
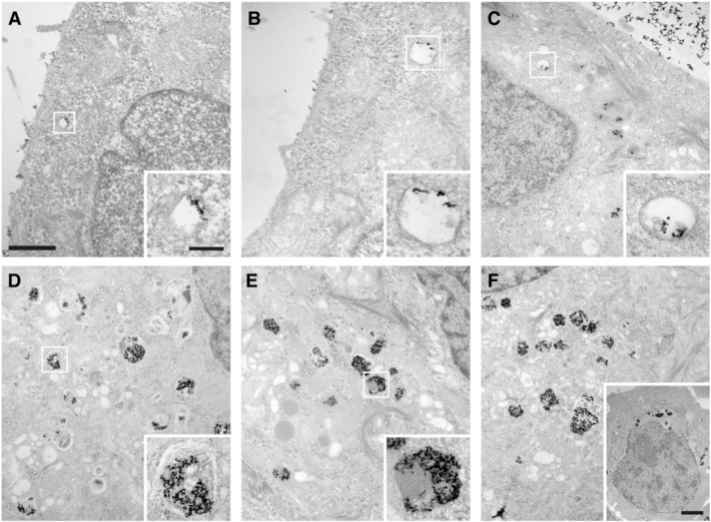
**Electron microscopy analysis of uptake kinetics.** Images from thin sections of MCF7 cells incubated with DMSA-SPION. **(a)** Cells incubated for 0.5 h, **(b)** 1 h, **(c)** 3 h, **(d)** 6 h, **(e)** 12 h and **(f)** 24 h. The inset in **(f)** shows the overall cell shape and morphology. Scale bars represent 1 μm for each image, 200 nm for insets in a to e, and 2 μm for the inset in f, respectively.

Samples from the same experiments were processed for observation by electron transmission microscopy (Figure 2). Even after very short incubation times (0.5 h), it was possible to detect SPION clusters within cell cytoplasm (Figure 2a). DMSA-SPION were found surrounded by a membrane and no free cytoplasmatic nanoparticles were detected. Incubations of 1 and 3 h revealed a small increment in the presence of vesicles containing DMSA-SPION (Figure 2b, c). During longer incubation times (6, 12 and 24 h), the number of vesicles with larger DMSA-SPION aggregates increased and they were accumulated close to the nuclei (Figure 2d-f and inset in f). Together with an increment in the number of vesicles, prolonged incubation time also resulted in important morphological changes of DMSA-SPION containing vesicles. While analysis of sectioned cells revealed a small increment in their size, the most important change however was related to their morphology, where a clear evolution from translucent vesicles with nanoparticles towards a much denser and multivesicular aspect has been detected (Figure 2 a-f).

As MCF-7 cells are derived from a human breast adenocarcinoma, we decided to study also DMSA-SPION uptake and accumulation in a non-malignant breast cell line MCF-10A. Cells were incubated with DMSA-SPION under the same conditions as MCF-7 cells. Analysis by bright field microscopy showed that uptake and accumulation of nanoparticles in MCF-10A cells was equivalent to MCF-7 cancer cells (see Additional file 1). This was confirmed by Prussian blue staining. Analysis by electron microscopy clearly revealed that aggregates of particles were accumulated inside MCF-10A cells near nucleus with similar kinetics to that found in carcinoma cells (Additional file 1). The overall response of these non-cancerous cells was similar to carcinoma cells (see Additional file 1).

Results obtained for nanoparticles internalization in malignant (MCF-7) and non-malignant (MCF-10A) cell lines are not entirely surprising. It is important to recall that all established cell lines, including non-malignant cells, have alterations in their genome, which make them different from healthy cells of an organism. Therefore, MCF-10A cannot be considered as a fully “normal” human cell line [25,26]. In this sense, quantum dot (QD) nanoparticles with different surface coatings can be internalized within human mammary non-tumorigenic epithelial cell line MCF-10A as well as in human mammary adenocarcinoma epithelial cell line MCF-7 [27]. Zhang *et al.* [28] have described that both (MCF-7 and MCF-10A) cells can internalize iron oxide nanoparticles by vesicular transport after incubation for different times (30 min, 4 and 24 h). This research was carried out using commercial iron oxide nanoparticles (maghemite γ-Fe_2_O_3_ with diameter around 30 nm) from Alfa Aesar® (Karlsruhe, Germany) without any coating.

In summary, it is rather difficult to compare our results with those reported in the literature previously, because nanoparticles used in other studies have very different characteristics. It is well known that parameters such as nanoparticle size and particle surface coating are crucial on nanoparticle-cell interactions [3,8,17,29].

### Internalization mechanism and accumulation of DMSA-SPION inside cells

**Figure 3:**
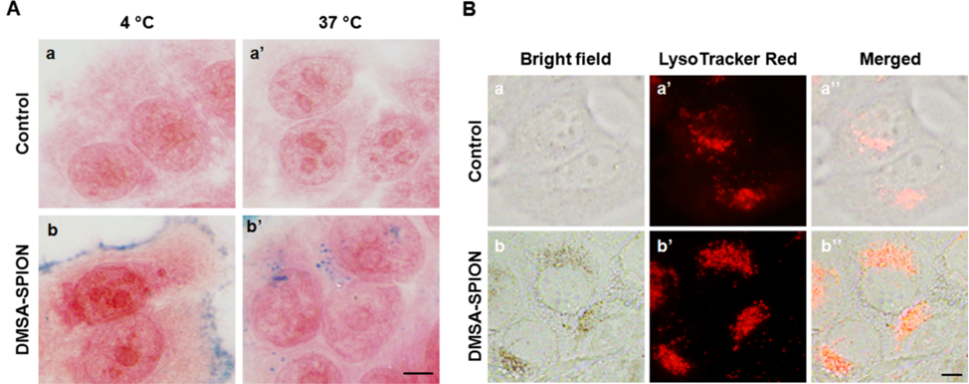
**Internalization mechanism and accumulation of DMSA-SPION inside cells. (A)** Temperature dependence of DMSA-SPION uptake. (a, a’) Control cells. (b) Cells incubated at 4 °C for 3 h with DMSA-SPION. (b’) Cells incubated for 3 h with same nanoparticles at 37 °C. Scale bars 10 μm. **(B)** Subcellular localization. (a, b) Visualization of control cells and cells incubated with nanoparticles for 24 h by bright field microscopy, respectively. (a’, b’) Lysosomes labeled with LysoTracker Red probe in the same cells, respectively. (a”, b”) Overlay images of control and treated cells, respectively. Scale bar 20 μm.

To analyze internalization mechanism, cells were incubated with particles at different temperatures. At 4°C, internalization of DMSA-SPION was inhibited and nanoparticles were attached at the cell surface, while uptake was developed successfully after 3 h at 37°C (Figure 3A). This result indicated that an active energy-dependent transport was implicated in the SPION internalization process [13,14,17,21].

To get insight into these nanoparticles subcellular localization, MCF-7 cells were incubated with DMSA-SPION for 24 h and then incubated with LysoTracker Red to stain the lysosomal compartment and finally visualized by bright field and fluorescence microscopy. Figure 3B show SPION into MCF-7 living cells using fluorescence microscopy. As can be seen in the same figure lysosomes were labeled with LysoTracker Red. Merged images displayed a substantial fraction of red fluorescence from LysoTracker which colocalizes with internalized nanoparticles, strongly suggesting that DMSA-SPION were accumulated in endosome/lysosome fraction.

**Figure 4:**
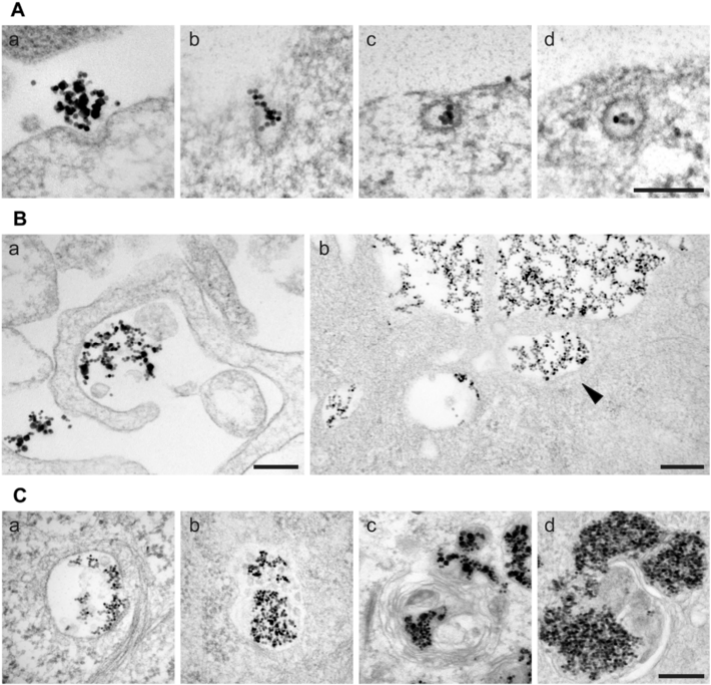
**Electron microscopy study of SPION interaction and uptake. (A)** Electron microscopy images of thin sections of cells interacting with DMSA-SPION by clathrin mediated uptake (<200 nm in diameter aggregates). Scale bar represents 200 nm. **(B)** Two images by electron microscopy of thin sections of cells showing typical images of macropinocytosis for DMSA-SPION uptake (>200 nm in diameter aggregates). Scale bars represent 200 nm. **(C)** Electron microscopy images of different types of endosomes containing SPION aggregates: (a) Early endosome. (b) Multivesicular body containing intraluminal vesicles. (c) Late endosome characterized by a multilamellar morphology. (d) Late endosomes and lysosomes with multivesicular structure and large electron-dense areas. Scale bar represents 200 nm.

To identify the precise mechanism of endocytosis (phagocytosis, pinocytosis, macropinocytosis, clathrin- mediated endocytosis, or caveolae-mediated endocytosis), we performed transmission electron microscopy (TEM) studies. The high contrast of the magnetic particles allowed for their clear identification (Figure 4). Small groups of particles were seen near cell membranes. Actually, SPION incubated in culture media present a relatively wide size distribution (ranging between 50 to more than 400 nm, see Additional file 2). Although we did not make an attempt to sort the SPION by size, we found significant differences in the way the SPION were incorporated in the cells according to the aggregate size. Smaller aggregates were seen adjacent to distinct clathrin-coated patches (Figure 4A). Closed clathrin vesicles containing small DMSA-SPION aggregates (smaller than 200 nm) were seen in the cytoplasm, near membrane. Larger DMSA-SPION aggregates were seen near cell periphery, in most cases engulfed by cell membrane extensions, indicating the existence of a macropinocytic DMSA-SPION uptake process (Figure 4B a, b). Other studies have also proposed a macropinocytic process for cationic iron oxide nanoparticles internalization [30], as well as for other nanoparticles [31].

Following short incubation times, particles were found near the cell membrane, showing SPION-containing vesicles closely resembling early endosomes (Figure 4C a). At later incubation stages, there were denser SPION-containing vesicles resembling multi-vesicular bodies containing intraluminal vesicles (Figure 4C b). Subsequently, the vesicles adopted a multi-lamellar lysosome aspect containing large numbers of DMSA-SPION clusters (Figure 4C c,d).

The same type of analysis has been carried out with the non-malignant MCF10-A cells. The results clearly showed that incorporation of DMSA-SPION and their intracellular trafficking feature the same overall characteristics in the case of the non-cancerous breast epithelial cells (see Additional file 1).

### Intracellular persistence of SPION

**Figure 5:**
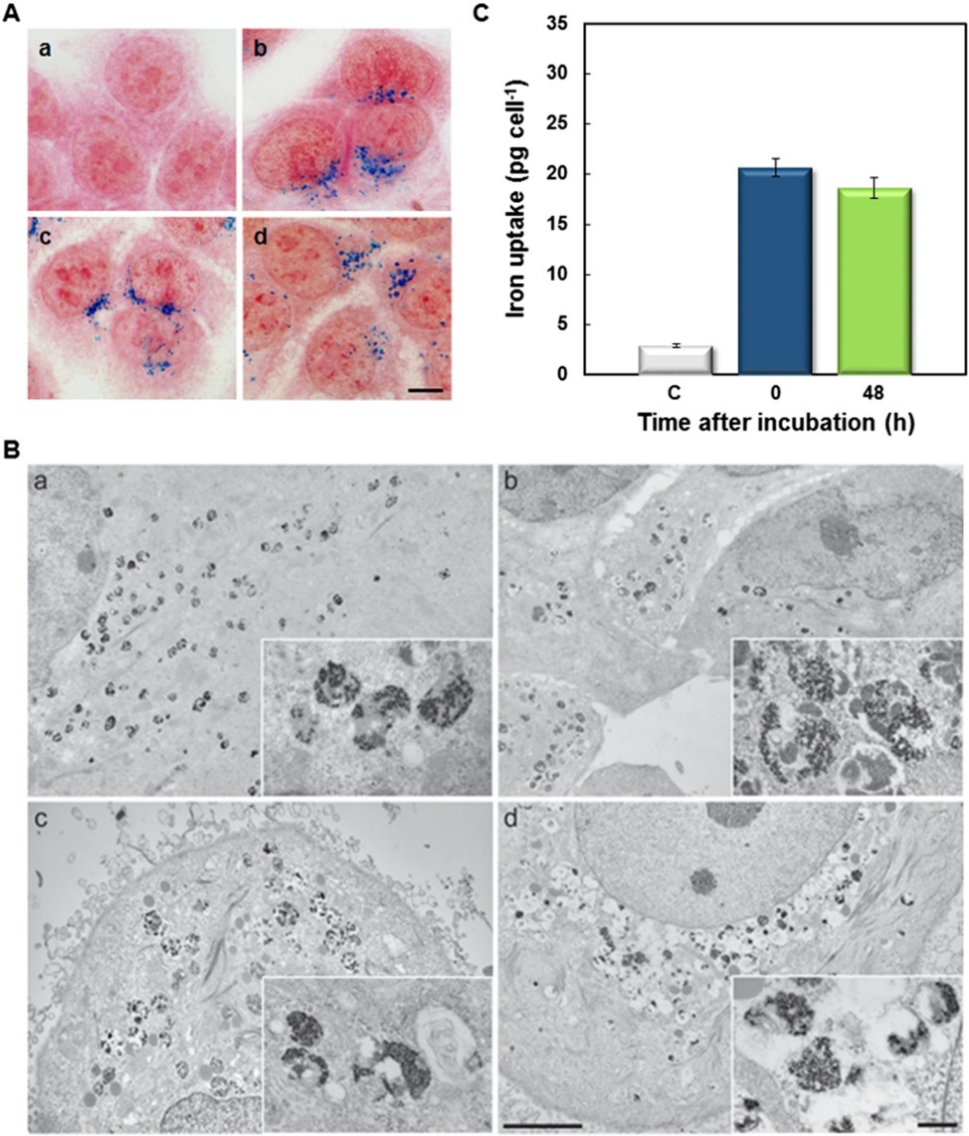
**Persistence of internalized DMSA-SPION. (A)** MCF-7 cells incubated with nanoparticles for 24 h, stained with Prussian blue reaction after different post-incubation times and visualized by bright field microscopy. (a) Untreated control cells. (b-d) Cells incubated for 24 h and stained 24, 48 and 72 h after incubation, respectively. Scale bar represents 10 μm. **(B)** Study of persistence by electron microscopy: (a) Cells were incubated with DMSA-SPION for 24 h. The cells were further incubated in medium without particles for additional (b) 24 h, (c) 48 h and (d) 72 h. Insets show larger magnification details of the endosomes. Scale bars represent 5 μm in overall areas and 500 nm in larger magnification insets, respectively. **(C)** Intracellular iron content quantification by ferrozine assay (expressed as weight of iron per cell) in control (non-treated) cells (c), and immediately (0) or 48 h after incubation with DMSA-SPION.

Other important questions related to the incorporation of nanoparticles into cells are to establish how long they remain inside cells and to disclose their eventual release mechanism. To get an insight into these questions, after 24 h incubation, nanoparticles were removed and cultures were further incubated up to 72 h at 37°C. Samples, taken at 24, 48 and 72 h, were stained with Prussian blue and observed by bright field microscopy. Figure 5A shows that SPION remain within MCF-7 cells in vesicles up to 72 h.

To get more detailed information on the evolution of the intracellular vesicles after prolonged incubation times, a parallel analysis to that described above was carried out using electron microscopy. Cells containing DMSA-SPION evolved and divided in a similar way as control cells without DMSA-SPION. Multi-vesicular bodies and lysosomes containing nanoparticles did not change much, even after extended incubation intervals (Figure 5B a-d). SPION clusters were retained inside the vesicles and these vesicles further evolved towards late endosomal or lysosomal morphology, but neither their number nor their localization in cell cytoplasm underwent significant changes, thereby indicating that DMSA-SPION were not massively released from cells. These results suggest that, although cells keep dividing, iron oxide nanoparticles persist inside them for a long time. These qualitative results were confirmed by quantification of intracellular iron content in ferrozine-based assay (Figure 5C), which confirmed that the amount of iron remains substantially unaltered inside the cells after 48 h post-incubation interval.

### Cytotoxicity of DMSA-SPION

Exposure to SPION has been associated with significant toxic effects due to the generation of ROS, which result in deleterious cellular consequences eventually leading to cell death [32-35]. There are contradictory results related to biocompatibility of DMSA-coated magnetic nanoparticles. Several reports have described some cytotoxicity for DMSA magnetic iron oxide nanoparticles in different cell lines [19,36]. On the contrary, little effects on cell viability, oxidative stress, cell cycle or apoptosis have been reported for these magnetic nanoparticles by other authors [17,37].

Taking into account such a contradictory background, we decided to analyze the biocompatibility of these nanoparticles using several complementary approaches, such as (i) studies of cytoskeletal components, (ii) cell morphology observations by bright field microscopy (neutral red and Hoechst-33258 staining), (iii) analysis of the cell cycle, (iv) detection of ROS generation and, (v) two alternative viability tests.

#### (i) Analysis of cytoskeletal components

**Figure 6:**
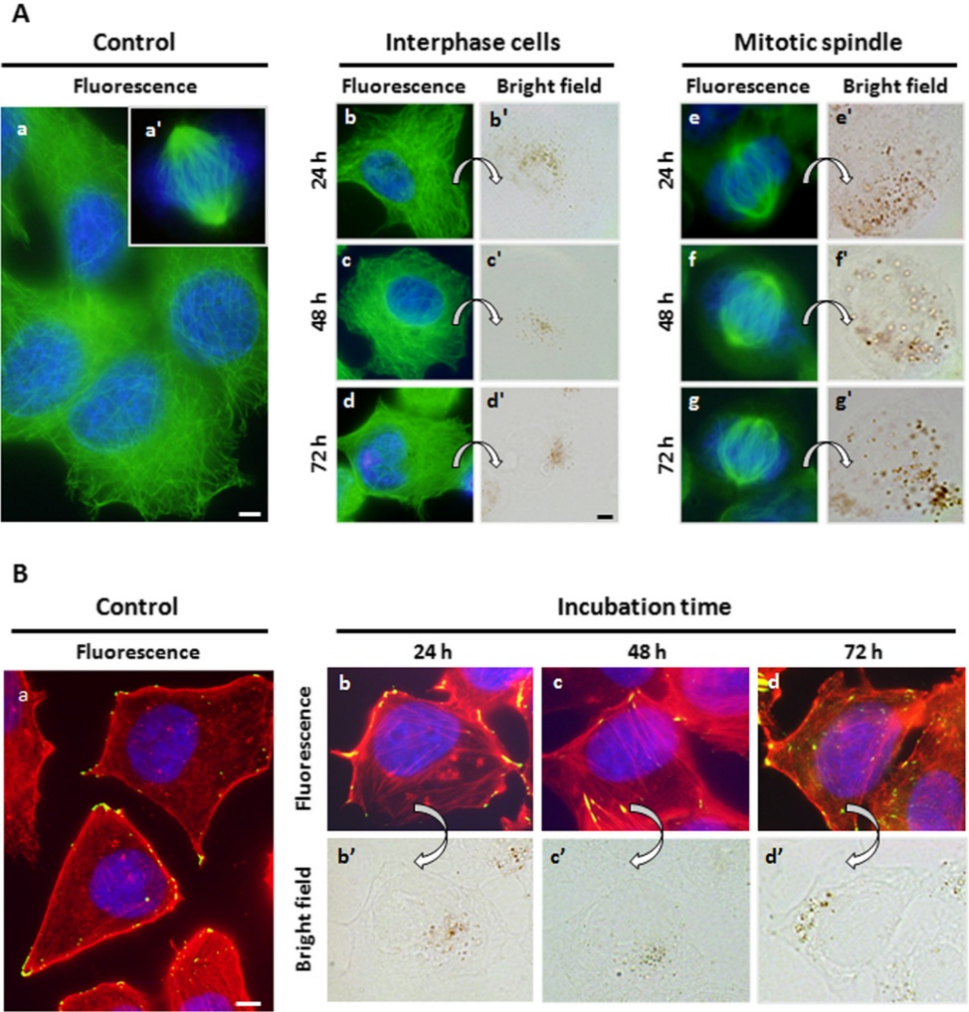
**Analysis of cytoskeleton. (A)** Representative images of cells immunostained for α-tubulin (green) and DNA counterstained with Hoechst-33258 (blue). (a) Interphase control cells. (a’) Metaphase control cell. (b-b’) Interphase cells incubated for 24 h with DMSA-SPION and observed by fluorescence and bright-field microscopy, respectively; (c-c’) cells incubated for 48 h; (d-d’) cells incubated for 72 h. (e-e’) Mitotic spindle of cells incubated for 24 h, (f-f’) 48 h and (g-g’) 72 h. **(B)** Merged images of F-actin labeled with rhodamine-phalloidin (red), vinculin immunostaining (green) and DNA counterstained with Hoechst-33258 (blue). (a) Control untreated cells. (b-b’) Cells treated with DMSA-SPION for 24 h and observed by fluorescence and bright-field microscopy, respectively. (c-c’) Cells incubated for 48 h. (d-d’) Cells incubated for 72 h. Scale bar 10 μm.

Two components of cytoskeleton were analyzed: microtubules (MTs) and actin filaments (F-actin). MTs are highly dynamic fibers of the cytoskeleton, with critical functions in eukaryotic cells including intracellular transport, organization of cell structural dynamics and cell division. We have evaluated the effects of nanoparticle internalization on MTs during interphase and mitosis by means of indirect immunofluorescence analysis to α-tubulin (DNA counterstained with Hoechst-33258). Figure 6A shows fluorescence images of MTs (green) and DNA (blue) for interphase and metaphase MCF-7 control cells. After 24, 48 or 72 h of incubation with nanoparticles, interphase microtubules maintain their normal morphology and distribution. In the same samples, DMSA-SPION were visualized inside the cells by bright field microscopy. Distributions of mitotic spindles and chromosomes were also similar to metaphase control cells up to 72 h after incubation.

We also investigated the effects of SPION on F-actin and vinculin, a protein implicated in cell adhesion as a focal adhesion complex component. F-actin builds the thinnest filaments of cytoskeleton in the cytoplasm of eukaryotic cells. They are involved in cell morphology, transport of vesicles and organelles, positioning of cellular components, cytokinesis, cell motion, cell-cell and cell-substrate interactions, and signal transduction. Focal adhesions are specialized sites containing a complex network of proteins, included vinculin, favoring interactions between cell and extracellular matrix through the actin cytoskeleton [38]. Figure 6B shows fluorescence images of actin microfilaments (red), vinculin protein (green) and DNA (blue) for MCF-7 control cells. Incorporation of DMSA-SPION, followed by localization of them inside the cells by bright field microscopy, did not affect the organization of stress fibers or focal adhesions.

#### (ii) Cell morphology

**Figure 7:**
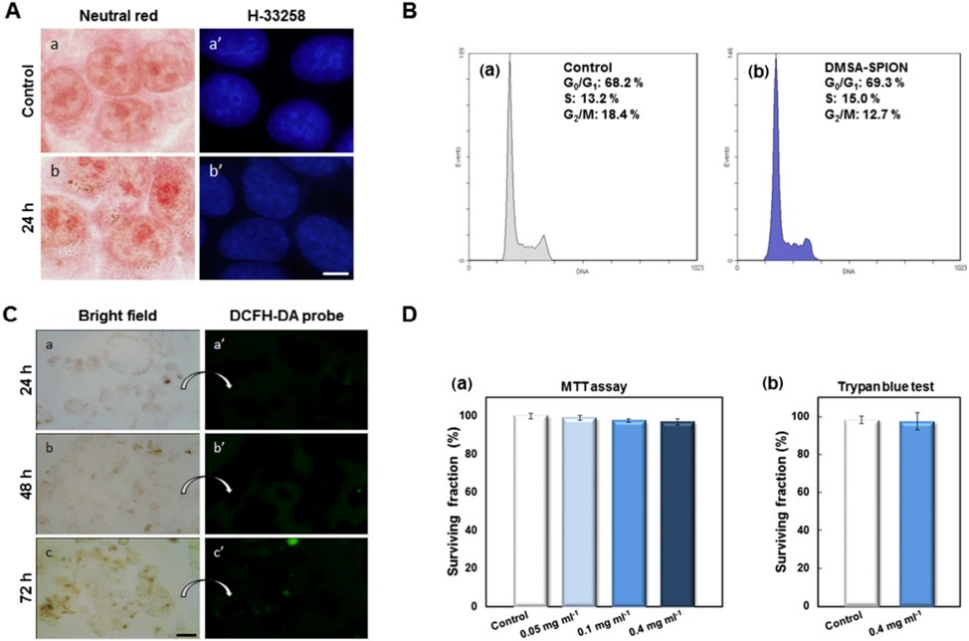
**Cytotoxic studies. (A)** Cell morphology by neutral red and Hoechst-33258 staining. (a-a’) Control cells. (b-b’) Cells incubated with DMSA-SPION for 24 h. Scale bar represents 10 μm. **(B)** Cell cycle analysis of control (untreated) and cells incubated with SPION for 24 h. **(C)** Analysis of ROS generation by DCFH-DA assay. Cells incubated with DMSA-SPION for different times and loaded with DCFH-DA were visualized under bright field or fluorescence microscopy, respectively. (a, a’) Cells incubated with nanoparticles for 24 h. (b, b’) Cells incubated for 48 h. (c-c’) Cells incubated for 72 h. Scale bar represents 50 μm. **(D)** Cytotoxicity analysis in MCF-7 cells incubated DMSA-SPION for 24 h. (a) MTT cell viability assay after 24 h of treatment with 0.05, 0.1 and 0.4 mg ml^−1^. (b) Trypan blue exclusion test immediately after incubation at 0.4 mg ml^−1^.

MCF-7 cells were exposed to 24 h incubation with DMSA-SPION and then they were stained with neutral red or Hoechst-33258 to observe cytoplasmic and nuclear morphology, respectively. Cells stained with neutral red have a similar morphology to control cells after nanoparticle incorporation in cytoplasm and, nuclei also presented the same characteristics as control cells (Figure 7A).

#### (iii) Cell cycle

As shown in Figure 7B, cell cycle distribution was not affected after incubation of cells with 0.4 mg ml^−1^ SPION for 24 h when compared to untreated control populations. A broad overview of the effects of magnetic and nonmagnetic nanoparticles on the cell life cycle has been recently compiled by Mahmoudi *et al.* [39]*.*

#### (iv) Detection of ROS generation

To analyze whether DMSA-SPION produce ROS, cells were preincubated with SPION for 24, 48 and 72 h and then treated with the fluorochrome probe DCFH-DA [40]. As shown in Figure 7C, no significant fluorescent signal was detected after 72 h incubation with SPION at 0.4 mg ml^−1^. Several *in vitro* studies have suggested that a range of iron oxide nanoparticles with different physico-chemical characteristics induce ROS formation which can lead to cellular injury and death [32-35].

#### (v) Cell viability studies

MTT assay showed that cell viability was not significantly affected by the presence of DMSA-SPION at 24 h of treatment (>96% viability in relation to the control sample), even at the highest concentration (0.4 mg ml^−1^) (Figure 7D a). The results obtained using Trypan blue assay (Figure 7D b), confirmed the biocompatibility of DMSA-SPION, and cell survival was > 90% after 24 h incubation. It is important to note that Trypan blue exclusion test has been proposed as the gold standard method to validate the cell viability after magnetic nanoparticle incubation [41]. These results were further confirmed using a multiparametric High Content Screening Cytotoxicity Assay in agreement with a previously published report [42], (data not shown).

In summary, the results presented here justify a deeper research on the synthesis and biological characterization of iron oxide nanoparticles. The complementary approaches recommended for risk assessment of nanoparticles [43,44] indicate that DMSA-SPION are safe and efficient nanoparticles for possible biomedical applications. This is a crucial fact, before further functionalization of these SPION for medical applications (drug delivery and/or hyperthermia), that require high levels of intracellular accumulation for effective treatment.

It is important to point out that the purpose of our study was twofold: i) to analyze the effectiveness of DMSA-SPION accumulation within tumor cells and ii) to confirm the absence of toxicity induced by nanoparticles (non-functionalized), to ensure their biocompatibility, even if they were accumulated by non-tumor cells. This is especially important, taking into account the pressing need to identify any potential cellular damage associated with SPION [32]. In the broader context, following such work carried out these “marginally toxic nanoparticles” will be further functionalized with biologically active molecular moieties such as peptides and antibodies for breast cancer targeting. From this prospective, our study is relevant to the safe development of nanoparticles for biomedical applications, as well as to understanding their biological behavior in the “bare” or non-functionalized state, since once delivered inside the cells, nanoparticles can be processed by intracellular pathways (e.g. distinct endocytic pathways) and “stripped” or separated from the molecules they have been originally conjugated with.

## Conclusions

Dimercaptosuccinic acid surface coating of SPION enhanced their cellular uptake efficiency without inducing either cytotoxicity, alteration of the major cytoskeletal components, vinculin protein dynamics, cell cycle or ROS formation in MCF-7 breast cancer cell line. Incorporation of DMSA-SPION inside the cells followed two endocytic pathways depending on the size of the particle aggregates: smaller aggregates were incorporated using a clathrin-dependent path, while larger aggregates were incorporated by macropinocytosis. In all cases, SPION aggregates were found surrounded by endocytotic membrane, which localized in perinuclear areas after long incubation times, but never inside the cell nucleus. Following cellular uptake, SPION showed a slow release rate and continuous persistence over extended intervals inside the cells. These characteristics are relevant for the rational design and subsequent utilization of SPION for biomedical applications, both for diagnosis by magnetic resonance imaging (MRI) and for targeted therapy of cancer by hyperthermia and releasing anti-cancer molecules with significantly reduced side effects.

## Methods

### Magnetic nanoparticles

Superparamagnetic iron oxide nanoparticles of uniform size (15 nm) were obtained by thermal decomposition of an iron oleate complex in 1-octadecene [12]. These particles, with a coating of DMSA that make them stable in aqueous buffers, were kindly provided by Dr. Puerto Morales (ICMM-CSIC) as part of MULTFUN FP7 NMP project (see details in Additional file 2).

DMSA-SPION were sterilized by 0.22 μm pore size filtration (Millipore Corp., Bedford, USA). SPION stock at 4 mg ml^−1^ was dispersed by sonication for 5 min in a 40 kHz sonicator bath (Branson 3510 ultrasonic cleaner, Thomas Scientific, Swedesboro, USA). SPION were then resuspended in complete cell culture media at a final concentration of 0.4 mg ml^−1^. The mixture was then sonicated for 1 min and incubated with cells at different times.

### Cell cultures

Human breast cancer MCF-7 cells were grown as monolayer cultures in Dulbecco’s modified Eagle’s medium (DMEM), supplemented with 10% (v/v) fetal bovine serum (FBS), 50 units ml^−1^ penicillin and 50 μg ml^−1^ streptomycin. All products were purchased from Gibco (Paisley, Scotland, UK) and sterilized by means of 0.22 μm filters. Cell cultures were grown in an incubator with 5% CO_2_ plus 95% air at 37°C. Depending on the purpose of experiment, cells were seeded on 24-well plates (with or without 10 mm square coverslips) or 25 cm^2^ flasks. Sub-confluent cell cultures were used. All sterile plastics were sourced from Corning (Corning Inc., New York, USA).

Non-tumorigenic human breast epithelial cell line MCF-10A was used for comparison in some experiments (see Supporting Information). Cell lines used in this study were obtained from American Type Culture Collection (ATCC)®.

### DMSA-SPION internalization

#### Live cell imaging

In order to analyze internalization of nanoparticles, MCF-7 cells were grown on coverslips and incubated for 24 h with DMSA-SPION. After incubation, culture medium was removed and samples were washed three times with phosphate-buffered saline (PBS, pH 7.4). Then, cells were observed immediately under bright light microscopy without being processed, to avoid potential fixation artifacts.

### Prussian blue staining

Cells preincubated with nanoparticles for different periods of time (0.5, 1, 3, 6, 12, 24, 48 or 72 h), were visualized by Prussian blue staining for iron detection [17,45]. Briefly, cells were fixed in methanol (at −20°C) for 5 min, stained with an equal volume of 4% hydrochloric acid and 4% potassium ferrocyanide trihydrate for 15 min, and counterstained with 0.5% neutral red for 2 min. Preparations were then washed with distilled water, air dried, and mounted in DePeX (Serva, Heidelberg, Germany). All other reagents were purchased from Panreac Química (Montcada i Reixac, Spain).

### Quantification of iron in cultured cells

#### Colorimetric ferrozine-based method

This sensitive assay permits the quantification of iron in cultured cells [46]. In time-dependent studies, MCF-7 cells seeded in 24-well plates were incubated with DMSA-SPION at a fixed concentration of 0.5 mg ml^−1^ for 24 or 48 h. For intracellular persistence studies, cells were incubated 24 h and intracellular iron content was evaluated 48 h after removing DMSA-SPION from culture media by three washes with PBS. After that, in both cases, cells in three wells were trypsinized and cell concentrations per well were determined by hemocytometer with 0.4% Trypan blue solution. Cells grown in other 24-well dishes were frozen at −20°C for 1 h and then, 500 μl of 50 nM NaOH (Panreac Química) were added to each well for 2 h in movement. Aliquots of cell lysates were then transferred to 1.5 ml eppendorf and mixed with 500 μl of 10 mM HCl, and 500 μl of iron-releasing reagent (a freshly mixed solution of equal volumes of 1.4 M HCl and 4.5% (w/v) KMnO_4_ (Merck, Germany) in distilled H_2_O. These mixtures were incubated for 2 h at 60°C within a fume hood, since chlorine gas is produced during the reaction. After the mixtures had cooled to room temperature, 150 μl of iron-detection reagent (6.5 mM ferrozine (Sigma-Aldrich, St Louis, USA), 6.5 mM neocuproine (Sigma-Aldrich), 2.5 M ammonium acetate (Panreac Química), and 1 M ascorbic acid (Sigma-Aldrich) dissolved in water) were added to each tube. After 30 min, 500 μl of the solution obtained in each tube was transferred into a well of a 24-well plate, and absorbance was measured at 570 nm in a SpectraFluor spectrophotometer (Tecan Group Ltd., Männedorf, Switzerland). Iron content of the sample was calculated by comparing its absorbance to that of a range of standard concentrations of equal volume, that had been prepared in a way similar to that of the sample (mixture of 100 μl of FeCl_3_ standards (0–300 μM) in 10 mM HCl, 100 μl 50 mM NaOH, 500 μl releasing reagent, and 1500 μl detection reagent). The determined intracellular iron concentration for each well of a cell culture was normalized against number of cells per well.

#### High content screening

Quantification of iron oxide content was based on automated epifluorescence images taken from stained cell monolayer cultured on slides. On average 100 cells were selected from the two cell line provided. Images were analyzed by single channel, filtered and threshold of each channel was identified. Composite rebuilt in order to identify localization of SPION against cellular staining. Filtering was applied on the red and blue filter in order to account for the SPION or the cell only.

### Endocytic mechanisms

In order to analyze the degree of involvement of the endocytic mechanisms in internalization of nanoparticles, cells were preincubated with nanoparticles for 3 h at either 4°C or 37°C, washed three times with PBS, and stained with Prussian blue technique (as described above).

### Subcellular localization of nanoparticles

To determine DMSA-SPION subcellular location inside cells, endocytic compartments of MCF-7 cells were labelled with 50 nM LysoTracker® Red DND-99 (Molecular Probes, Eugene, Oregon, USA) fluoroprobe in culture medium, at 37°C for 30 min. Cells incubated with nanoparticles for 24 h were labelled with LysoTraker Red and then coverslips were washed with PBS and cells were observed immediately under bright field and fluorescence microscopy.

### Analysis of the cytoskeleton and adhesion proteins

#### Immunofluorescence staining of α-tubulin

Cells grown on glass coverslips were incubated with nanoparticles for 24, 48 and 72 h and then, immunostained for α-tubulin. Briefly, cells were fixed with cold methanol for 5 min, washed three times for 5 min with PBS, and then permeabilized with 0.5% Triton X-100 (Sigma-Aldrich) in PBS for 5 min. After Triton removal, cells were incubated with primary monoclonal mouse anti-α-tubulin antibody (Sigma-Aldrich) diluted 1:100 at 37°C in a wet chamber for 1 h. Three washings with PBS were then carried out before addiction of Triton X-100 for 5 min. Incubation of the secondary antibody (Fab specific goat anti-mouse FITC-IgG; Sigma-Aldrich) was identical to that of the first one. Then, DNA was counterstained by Hoechst-33258 (0.05 mg ml^−1^ in distilled water) for 5 min. Finally, cells were washed with PBS and mounted with ProLong Gold (Molecular Probes) antifade reagent.

#### Vinculin immunofluorescence and F-actin staining

For vinculin immunostaining, cells grown on coverslips were fixed with formaldehyde in PBS (1:10 v/v), for 20 min at 4°C, washed three times for 5 min with PBS and permeabilized with 0.5% Triton X-100. After incubation with a blocking solution (5% bovine serum albumin, 5% FBS, 0.02% Triton X-100 in PBS) at room temperature for 30 min, cells were incubated with 1:50 solution mouse monoclonal anti-vinculin (Sigma-Aldrich) at 37°C in a wet chamber for 1 h. Primary antibody binding was detected using Fab specific goat anti-mouse FITC-IgG diluted 1:50. F-actin was visualized in the same samples by incubation with rhodamine-labeled phalloidin (Sigma-Aldrich) diluted 1:200 at 37°C in a wet chamber for 25 min. Then, samples were washed three times with PBS, counterstained with Hoechst-33258 for 5 min, washed with PBS and mounted with Prolong Gold antifade reagent.

### Cell morphology analysis

#### Neutral red staining

MCF-7 cells grown on coverslips in 24-well plates were incubated with DMSA-SPION for 24 h, fixed in methanol at −20°C for 5 min and then stained with 0.5% neutral red for 2 min. After that, samples were washed with distilled water, air dried, mounted in DePeX and visualized by light microscopy.

#### Hoechst-33258 staining

Cells seeded on coverslips and treated with nanoparticles for 24 h were fixed in methanol at −20°C for 5 min and stained with Hoechst-33258 for 5 min. Samples were washed with distilled water, air dried, and mounted in DePeX for observation using fluorescence microscopy.

### Cell cycle analysis

MCF-7 cells were plated in 25-cm^2^ flasks and incubated with DMSA-SPION for 24 h. Analysis of cell cycle was performed by flow cytometry using propidium iodide (PI) labeling of DNA cell content. Cells were trypsinized (also harvesting possible detached cells) and centrifuged at 1200 rpm for 5 min. After centrifugation, pellet was resuspended in 100 μl of culture medium without phenol red. Then, it was added 50 μl of Coulter DNA Prep Reagents Kit (Beckman-Coulter Inc, California, USA), 1 ml of PI solution with RNase and incubated for 30 min at 37°C. Both reagents were purchased from Sigma-Aldrich. Distribution of cells in different phases of cell cycle was determined using a Coulter Epics XL-MCL flow cytometer (Beckman-Coulter Inc.) with an argon laser line (488 nm), complemented with appropriate filters, and a minimum of 10^4^ labeled cells per sample were analyzed in each experimental condition. Percent of cells in each phase of the cell cycle was compared with that of control cells (without nanoparticles incubation). At least 10000 fluorescent events were counted per sample.

### Measurement of intracellular ROS

Intracellular ROS levels were determined using 2′,7′-dichlorodihydrofluorescein diacetate (DCFH-DA) assay. Cells were seeded on coverslips and, after exposure to nanoparticles for 24, 48 and 72 h, were washed with PBS and incubated with 10 μM DCFH-DA (Sigma-Aldrich) for 30 min. Then, cells were washed with PBS again and visualized immediately by fluorescence microscopy. Bright field microscopy was also used to corroborate accumulation of nanoparticles. For control induction of oxidative stress, cells were treated with 800 μM H_2_O_2_ (Panreac Química) for 1 h 30 min in complete medium. ROS production was observed in cells, 1 h 30 min after that H_2_O_2_ was removed, with 10 μM DCFH-DA for 30 min.

### Cytotoxicity assays

#### MTT test

Cytotoxicity was assessed by MTT colorimetric assay 24 after incubation with DMSA-SPION. Immediately prior to use, a stock solution of dimethylthiazolyl-diphenyl-tetrazolium bromide (MTT; Sigma-Aldrich, 1 mg ml^−1^) in PBS was prepared. Five hundred microliters of this MTT solution (50 μg ml^−1^ MTT in culture medium) was added to each culture dish without coverslip. Cells were incubated for 3 h, then reduced formazan was extracted with 500 μl dimethylsulfoxide and absorbance measured at 570 nm in a SpectraFluor spectrophotometer (Tecan Group Ltd, Männedorf, Switzerland). Cell survival was expressed as the percentage of absorption of treated cells in comparison with that of control cells.

#### Trypan blue exclusion test

Cell viability was quantified by Trypan blue dye exclusion method. Briefly, after 24 h of incubation with DMSA-SPION, trypsin was added to control and treated cells. After cells were detached from the plate, they were resuspended in culture media. Equal volumes of each cell suspension and trypan blue solution (0.2% in PBS) were mixed and used for cell counting by hemocytometer. Blue-stained cells were counted as nonviable cells and unstained cells as viable cells.

### Bright field and fluorescence microscopy

Observation of samples processed for bright field and fluorescence microscopy were made with an Olympus BX61 epifluorescence microscope, equipped with an Olympus DP50 digital camera (Olympus, Tokyo, Japan), and processed using the Adobe Photoshop 7 software (Adobe Systems, San Jose, CA, USA). The following filters were used to visualize the fluorescence signal of probes: UV excitation light (365–390 nm) for Hoechst-33258, blue (460–490 nm) for FITC, and green (510–550 nm) for TRITC.

### Statistical analysis

Statistical analysis was performed by GraphPad Prism Software (GraphPad Inc., CA, USA) using one-way ANOVA and Tukey’s test. The threshold for significance was *P* = 0.05 and *P* values < 0.05 (_*_), < 0.01 (_**_) and < 0.005 (_***_) were considered as significant.

### Sample preparation for transmission electron microscopy

MCF-7 cells were incubated with SPION at different times, as described above, washed with PBS and treated with a mixture of 2% formaldehyde (Ultra Pure EM Grade, Polysciences Inc., Philadelphia, USA) and 2.5% glutaraldehyde (EM Grade, TAAB Laboratories Equipment Ltd., Berks, UK) in PBS for 1 h at room temperature. The cell monolayer on the coverslips was then washed with PBS and distilled water, post-fixed for 45 minutes with 1% osmium tetroxide (TAAB Laboratories Equipment Ltd.) in PBS, washed with distilled water, treated during 45 minutes with 1% aqueous uranyl acetate (Electron Microscopy Sciences, Hatfield, USA), washed again and dehydrated with growing quantities (50%, 75%, 95% and 100%) of ethanol seccosolv (Merck KGaA, Darmstadt, Germany). The samples were maintained in coverslips throughout the process and finally embedded in epoxy resin 812 (TAAB Laboratories Equipment Ltd.) contained in gelatine capsules (Electron Microscopy Sciences). The epoxy resin was polymerized for 2 days at 60°C. Resin was detached from the coverslips by successive immersions in liquid nitrogen and hot water. Ultrathin, 70-nm-thick sections were obtained with an Ultracut UCT ultramicrotome (Leica Microsystems), transferred to 200 mesh Nickel EM grids (Gilder, Lincolnshire, UK) and stained with 3% aqueous uranyl acetate (10 minutes) and lead citrate (2 minutes) (Electron Microscopy Science). Sections were visualized on a JEOL JEM 1200 EXII electron microscope operating at 100 kV (JEOL Ltd., Tokyo, Japan).

## Additional files

Additional file 1:
**Uptake, accumulation and cytotoxicity of DMSA-SPION into non oncogenic MCF-10A cells.**
Additional file 2:
**Nanoparticle concentration and stability characterization by Nanoparticle Tracking and Analysis (NTA).**

## Abbreviations

SPION: Superparamagnetic iron oxide nanoparticles
DMSA: meso-2,3-dimercaptosuccinic acid
DMSA-SPION: Dimercaptosuccinic acid coated superparamagnetic iron oxide nanoparticles
MNP: Magnetic nanoparticles
NP: Nanoparticles
TEM: Transmission electron microscopy
PBS: Phosphate buffered saline buffer
FBS: Fetal bovine serum
DMEM: Dulbecco’s modified Eagle’s medium
HCl: Hydrochloric acid
MTs: Microtubules
F-Actin: Actin filaments
FITC-IgG: Fluorescein isothiocyanate conjugated immunoglobulin-G
ROS: Reactive oxygen species
DCFH-DA: 2′,7′-dichlorodihydrofluorescein diacetate
PI: Propidium iodide
MRI: Magnetic resonance imaging
MTT: Dimethylthiazolyl-diphenyl-tetrazolium bromide
