# Supplementary material for: Characterization of interaction of magnetic nanoparticles with breast cancer cells
Source: J Nanobiotechnology. 2015 Feb 26;13:16. doi: 10.1186/s12951-015-0073-9 (PMC4403785; doi:10.1186/s12951-015-0073-9)
Supplement: Additional file 1: — Uptake, accumulation and cytotoxicity of DMSA-SPION into non oncogenic MCF-10A cells. [file 12951_2015_73_MOESM1_ESM.doc]

# Additional files

### Additional file 1 –Uptake, accumulation and cytotoxicity of DMSA-SPION into non oncogenic MCF-10A cells

As MCF-7 cells are derived from a human breast adenocarcinoma, we decided to study DMSA-SPION uptake and accumulation in a control, non-carcinoma breast cell line. MCF-10A Basal B subtype cell line model is considered normal-like as it is non-oncogenic and is derived from a fibrocystic disease. It provides a measure of effectiveness with regards healthy cell line versus result obtained in MCF-7 cells.

**Cell culture**

MCF-10A cells were maintained in high glucose Dulbecco's Modified Eagle's Medium/Ham's F12 50:50 mixture supplemented with 5% horse serum, Epidermal Growth Factor (100 µg ml^-1^), Hydrocortisone (1 mg ml^-1^), Cholera Toxin (1 mg ml^-1^), Insulin (10 mg ml^-1^) and Penicillin/Streptomycin (500 µl, 10000 U ml^-1^ penicillin and 10 mg ml^-1^ streptomycin), which were all purchased from Sigma-Aldrich.

**Uptake and accumulation**

Several analyzes were performed in MCF-10A non-tumorigenic cells after incubation with DMSA-SPION (0.4 mg ml^-1^) for 24 h: i) Analysis of labeling efficiency by Prussian blue staining showed that uptake and accumulation of nanoparticles in MCF-10A cells was equivalent to MCF-7 cancer cells (Figure S2A); ii) Mechanism of cellular uptake by electron microscopy analysis revealed that aggregates of particles accumulated in MCF-10A cells near the nucleus with similar kinetics to that found for carcinoma cells (Figure S2B); iii) Cell morphology observations by optical microscopy (neutral red and Hoechst-33258 staining). Neutral red staining shows that cells with nanoparticles inside have a similar morphology to control cells, and nuclei have the same characteristics in both cases (Figure S2C); iv) Determination of ROS production. DMSA-SPION containing cells did not generate ROS (Figure S2D).

**~~
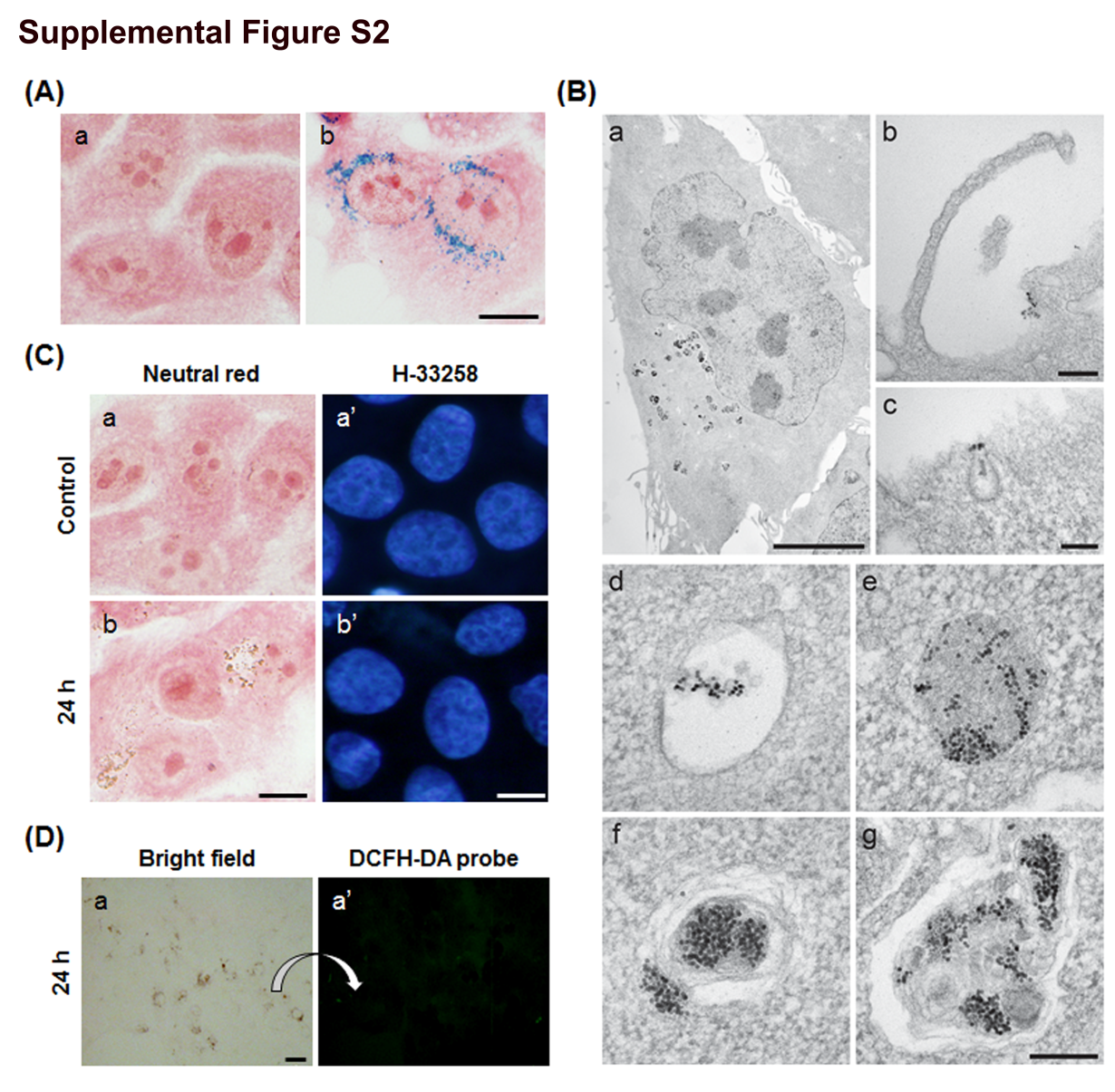
~~**

**Supplemental Figure S1** **Uptake and accumulation of DMSA-SPION into non-oncogenic MCF-10A breast cells**. **(A)** Cells incubated with DMSA-SPION and stained with Prussian blue reaction. (a) Control cells. (b) Cells incubated with 0.4 mg ml^-1^ DMSA-SPION for 24 h showed blue spots located inside cells. Scale bar 10 µm. **(B)** SPION distribution inside MCF-10A cells by electron microscopy. (a) Large overview of a cell revealing several aggregates of high contrast SPION aggregates in the nuclear periphery. Scale bar represents 5 µm. (b) Macropinocytosis of a group of SPION. Scale bar represents 200 nm. (c) A small group of nanoparticles in contact with the membrane of the cell inducing the formation of a clathrin-coated pit. Scale bar represents 100 nm. (d) Early endosome with clathrin lattice and a few ILVs (intraluminal vesicles). (e) Lysosome, with electron dense lumen. (f) LE (Late endosome) showing characteristic multilamellar aspect. (g) LE and lysosome showing multilamellar and multivesicular structures with electron dense areas, consistent with the existence of degradative processes. Scale bar in (d) to (g) represents 200 nm. **(C)** Cell morphology by neutral red and Hoechst-33258 staining. (a-a’) Control cells. (b-b’) Cells incubated with DMSA-SPION. Scale bar represents 10 μm. D) Qualitative characterization of ROS generation by DCFH-DA assay. (a, a’) Cells incubated with nanoparticles for 24 h and loaded with DCFH-DA visualized under bright field and fluorescence microscopy, respectively. Scale bar 50 µm.

**Cytotoxicity**

Nanoparticle cytotoxicity to MCF-10A cells was investigated using the MTT assay.

*
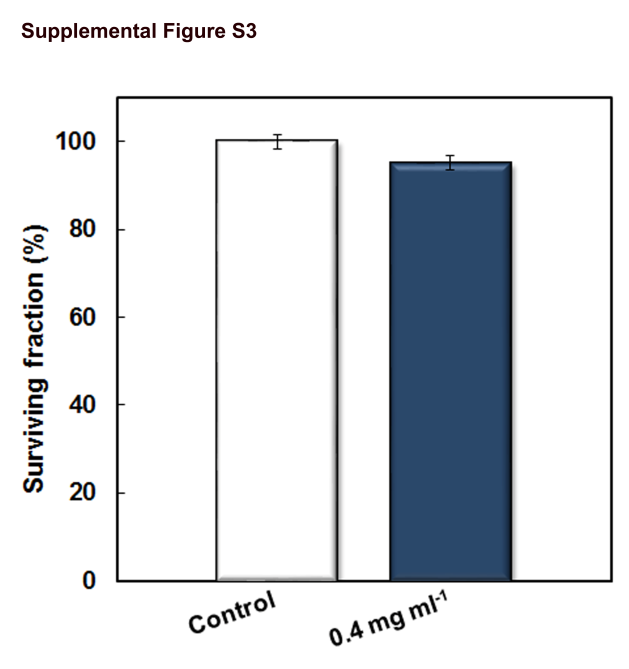
*

**Supplemental Figure S2** **MTT assay**. Results from MTT assay indicated that DMSA-SPION have no effects on MCF-10A cell viability.
