# Supplementary material for: Characterization of interaction of magnetic nanoparticles with breast cancer cells
Source: J Nanobiotechnology. 2015 Feb 26;13:16. doi: 10.1186/s12951-015-0073-9 (PMC4403785; doi:10.1186/s12951-015-0073-9)
Supplement: Additional file 2: — Nanoparticle concentration and stability characterization by Nanoparticle Tracking and Analysis (NTA). [file 12951_2015_73_MOESM2_ESM.doc]

# Additional files

### Additional file 2 – Nanoparticle concentration and stability characterization by Nanoparticle Tracking and Analysis (NTA).


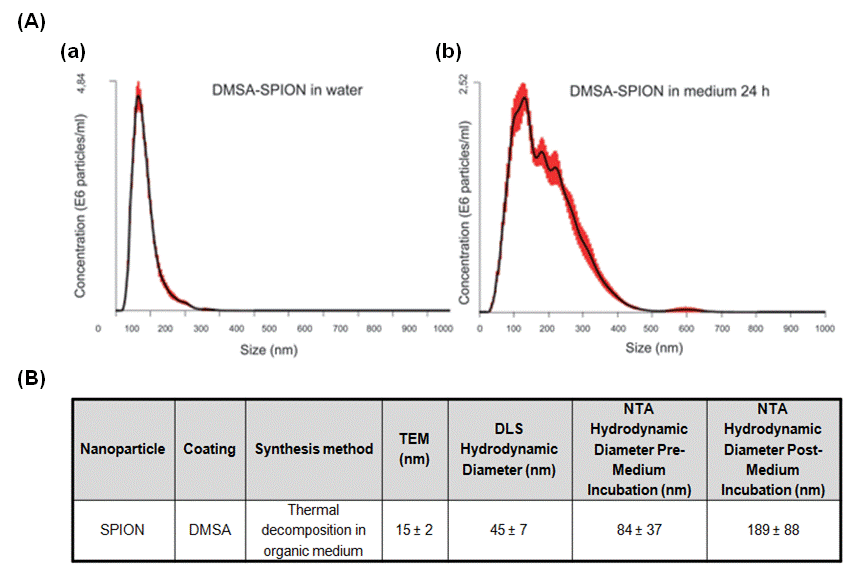
DMSA-SPION used in this study was fully characterised using the NTA technique. Physical characteristics from size and polydispersity were determined in a range of biological mediums. DMSA-SPION nanoparticles were diluted to 1 μg ml^-1^using either 0.22 μm filtered pH 7.4 DEPC water (Invitrogen, UK) or 0.22 μm filtered DMEM medium containing 10% FBS (Sigma Aldrich). Samples were incubated for 24 h at 37 °C in a humidified environment containing 5% CO_2_ and analysed using NTA. The Nanosight NS500 microfluidic instrument (Nanosight, Amesbury, UK) was used to identify and analyse individual MNPs to generate size and dispersity. The device consists of an EMCC camera (ANDOR, Belfast, UK) mounted on a conventional optical microscope with a 20x objective and LM14 viewing unit containing a 532 nm continuous wave laser light source. Introduction of each sample into the LM14 viewing unit was automated via on board peristaltic pump with manual focus of nanoparticle following the manufacturer’s standard operating procedures. The NanoSight NS500 recorded six independent 90 second videos containing fresh nanoparticle populations with each recording. Analysis was conducted in batch mode and analysed with the NTA 2.3 software.

**Supplemental Figure S3** **Nanoparticle Tracking Analysis of DMSA-SPION**. **(A)** Size distribution and response when dispersed (a) in water and (b) in DMEM medium solution after 24 h incubation. **(B)** DMSA-SPION fingerprint property table included as a summary.
